# On the trail of a critically endangered fungus: A world-first application of wildlife detection dogs to fungal conservation

**DOI:** 10.1016/j.isci.2024.109729

**Published:** 2024-04-29

**Authors:** Michael D. Amor, Shari Barmos, Hayley Cameron, Chris Hartnett, Naomi Hodgens, La Toya Jamieson, Tom W. May, Sapphire McMullan-Fisher, Alastair Robinson, Nicholas J. Rutter

**Affiliations:** 1 Royal Botanic Gardens Victoria, Melbourne, VIC 3004, Australia; 2 Department of Aquatic Zoology, Western Australian Museum, Welshpool, WA 6106, Australia; 3 Centre for Geometric Biology, School of Biological Sciences, Monash University, Clayton, VIC 3800, Australia; 4 School of BioSciences, University of Melbourne, Parkville, VIC 3052, Australia; 5 Zoos Victoria, Parkville, VIC 3052, Australia

**Keywords:** Canine behavior, Nature conservation, Ecology

## Abstract

Plant and animal conservation have benefited from the assistance of wildlife detection dogs (WDDs) since 1890, but their application to fungal conservation has not been trialed. In a world-first, we tested the effectiveness of WDDs and human surveyors when searching for experimentally outplanted fungi in natural habitat. We focused on a critically endangered fungus from Australia, *Hypocreopsis amplectens,* and showed that a WDD outperformed a human surveyor: our WDD detected a greater proportion of targets, had a faster time to first discovery, and had fewer false negatives. Our study highlights the tremendous potential for WDDs to enhance fungal conservation by demonstrating their utility in one of the most challenging fungal systems: a rare species with low population densities and low volatility. Our findings suggest that the application of WDDs to fungal conservation should enhance continuing efforts to document and conserve an understudied kingdom that is threatened by habitat loss and climate change.

## Highlights

• Novel application of wildlife detection dogs to fungal conservation
• Our dog outperformed our most skilled human in a challenging fungal system
• Our dog team found more targets, in less time, with fewer false negatives
• We extend the application of wildlife detection dogs to an overlooked kingdom

## Graphical abstract

**Figure:**
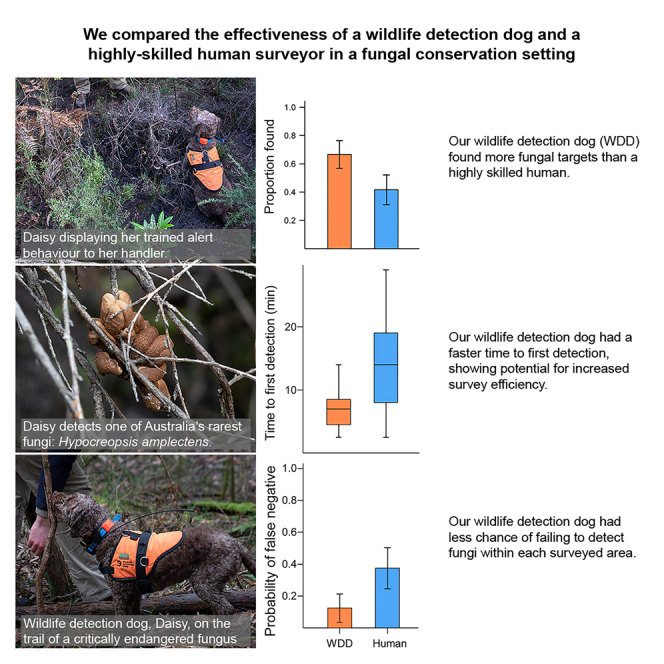

## Introduction

Conservation of threatened species is underpinned by our ability to detect them—that is, to accurately determine their presence or absence in an area. For example, to be listed on the International Union for the Conservation of Nature’s (IUCN) Red List of Threatened Species, the distribution, abundance, and number of mature individuals of a species must be reliably known.^1^ Yet uncertainty and inaccuracy of data for these parameters may arise for several reasons, such as: the inherent rarity of vulnerable organisms; ambiguous distributions and/or suitable habitat; difficulty accessing areas deemed suitable for survey; inadequate resourcing/survey effort; small size of individuals; and crypsis.

To overcome detection-related issues, we often rely on technology for assistance.^2^ Camera traps and auditory sensors, for example, are utilized to confirm the presence of certain species within an area, but they cannot detect sessile or non-vocal targets, respectively. Environmental DNA (eDNA) markers can identify species from environmental samples, overcoming some of these limitations.^3^ However, the above approaches do not provide a means of detecting species in real-time and, therefore, do not allow for immediate sampling for further investigation. As such, potentially valuable conservation measures cannot be carried out in real-time, such as the collection of individuals for genetic sampling, breeding programs, or health assessments.

Wildlife Detection Dogs (WDDs) have assisted humans with the conservation of threatened species since at least 1890,^4^ in applications ranging from controlled laboratory conditions (e.g., Seal^5^) to field-based detection in real-time. Relative to humans, WDDs demonstrate a greater ability to cover large areas and detect elusive, low-density^6^^,^^7^^,^^8^ and near-invisible targets.^9^^,^^10^^,^^11^ WDDs have an adaptive skillset and flexible detection criteria, with past studies demonstrating that training can be optimized for the identification of a specific individual^12^ through to a broad target group (e.g., detecting across species and genera;^13^^,^^14^). WDDs can also be trained to detect multiple targets, allowing them to work effectively within habitat shared by sympatric threatened taxa (e.g., Long et al.^15^, Vynne et al.^16^, Wasser et al.^17^).

Perhaps unsurprisingly then, WDDs have assisted with the conservation of many plants and animals (reviewed in Beebe et al.^18^, Grimm-Seyfarth et al.^19^) and in 85% of cases (n = 422), they outperformed humans or other methods of detection.^18^ However, their involvement in fungal detection has been limited to locating fungi of significance to agriculture and forestry, such as wood-decay and pathogenic fungi, along with fungi utilized as food sources.^19^ In these applications, WDDs excel. For example, WDDs have aided the detection of gourmet truffles for centuries,^20^ and are highly effective at detecting pathogenic fungi in agricultural settings with ≥97% accuracy.^21^ Despite their demonstrated potential for finding fungal targets, however, to our knowledge, WDDs have not yet been incorporated into fungal conservation efforts.

Fungi are a vital component of nutrient cycling, they form key associations to enhance plant productivity and resilience, and they provide food and medicinal compounds.^22^^,^^23^^,^^24^ Fungal conservation is therefore vital to human well-being and the health of our planet. However, this kingdom is severely understudied, with only 5% of the estimated 2–4 million species described.^25^ Of these described species, only 597 have had their conservation status documented on the IUCN Red List—nearly half of which (48%) are classified as vulnerable, endangered, or critically endangered.^26^ Fungal conservation is clearly in its infancy and, given their ecological and economic value, finding new ways to enhance fungal conservation is sorely needed.^27^

**Figure 1:**
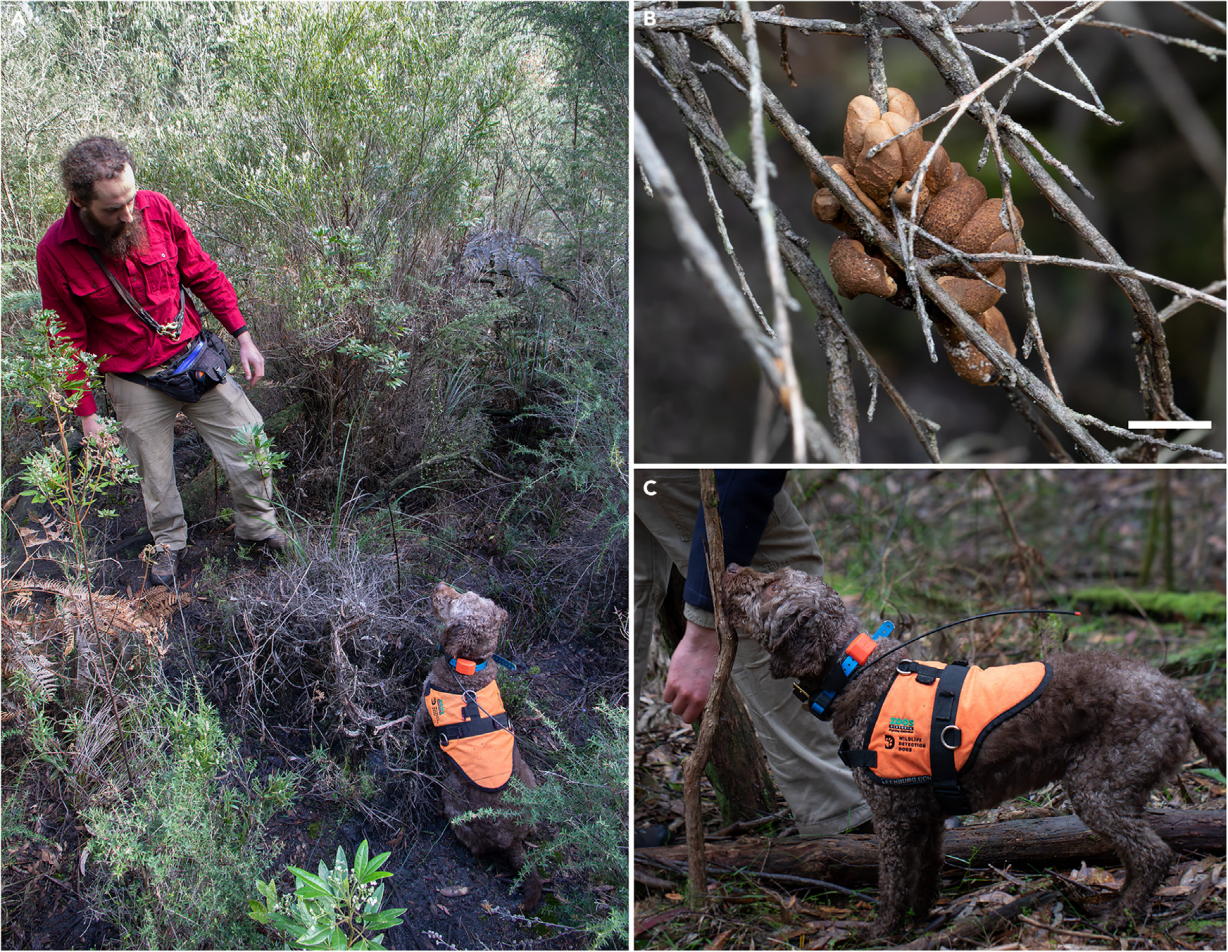
Wildlife Detection Dog, Daisy, on the trail of *Hypocreopsis amplectens* (A) Daisy displaying her trained alert behavior to her handler after detecting a naturally occurring specimen *in situ*. (B) The specimen discovered by Daisy in A. Scale, 10 mm. (C) Profile photograph of Daisy during an *in situ* training session.

Our study provides the first test of how effectively WDDs can aid fungal conservation. We focus on one of Australia’s rarest fungi, tea-tree fingers (TTF; *Hypocreopsis amplectens*), which is currently listed as Critically Endangered (CR) on the IUCN Red List.^28^ TTF is a mycoparasite and thus relies on the presence of another fungus as a food source.^29^ Further, TTF has an extremely limited distribution, with <100 specimens known from six discrete sites in south-eastern Australia. Like many fungi, TTF has been severely impacted by habitat loss and fragmentation, thus the protection of known sites and the detection of new sites is essential to safeguard against further loss. However, the visual detection of TTF is extremely difficult, as it occurs at low densities within complex vegetation (Figure 1A) and is highly cryptic: individual sporing bodies are small (to 140 mm long but often smaller), brown (similar in color to the underlying wood) and stalkless. Habitat surveys are therefore extremely time consuming and difficult, such that WDDs could greatly assist survey efforts for this rare and threatened species.

Here, we assessed the effectiveness of a WDD for the *in situ* detection of TTF specimens in a natural habitat and compared their performance to that of a human surveyor. We conducted an experiment where we outplanted a known number of TTF specimens into plots at three sites, and compared detection rates, the time taken to first detect an outplanted specimen, false positives, and the probability of incorrectly determining the absence of TTF in a plot where it had been outplanted (i.e., false negative rates). We also compared the effectiveness of WDDs and human surveyors in detecting naturally occurring individuals *in situ*.

## Results

### Detection of experimental outplants

#### Proportion of outplants detected

**Figure 2:**
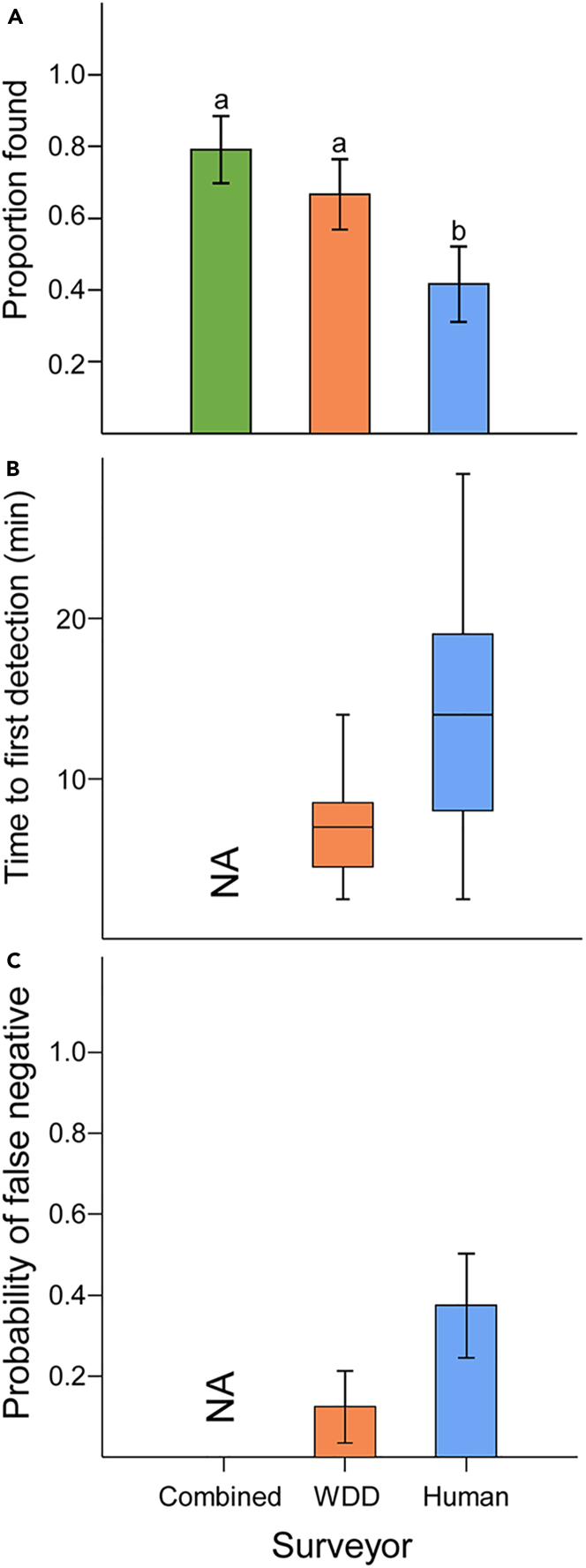
Comparison of wildlife detection dog (WDD), human, and combined performance when searching for experimentally outplanted tea-tree fingers (*Hypocreopsis amplectens*) (A) Proportion of experimental outplants detected. Letters above bars show Tukey’s post hoc test results. (B) Boxplots summarizing time to first detection. Median values (horizontal) and interquartile ranges (vertical) are shown. (C) Probability of failure to detect TTF within a plot if one or more outplanted specimens were present (i.e., false negatives). Error bars (A,C) represent standard error.

We found a significant difference in the proportion of outplanted TTFs detected by surveyor type (F = 7.621, *df* = 2,30, *p* = 0.002; Figure 2). The combined tally of human and WDD-team search efforts yielded higher detection rates relative to the efforts of a human surveyor alone (Tukey’s HSD test: t = −0.375; *p* = 0.001; CI95% = -0.606, −0.148), but was equivalent to detection rates of the WDD-team alone (Tukey’s test: t = −0.125; *p* = 0.408; CI95% = -0.356, −0.1). Our WDD-team detected a greater proportion of outplants than our human surveyor (Tukey’s test: t = −0.25; *p* = 0.029; CI95% = -0.481, −0.024). There was no effect of site on outplant detection (F = 0.047, *df* = 2, 13, *p* = 0.955).

#### Time to first detection

Our WDD-team had faster time to first detection relative to our human surveyor—once we excluded a single outlier plot from our analysis (F = 7.074, *df* = 1, 10, *p* = 0.023). This plot was characterized by very dense vegetation, and our dog-handler communicated that Daisy was showing signs of fatigue. In this plot, Daisy had an uncharacteristically long time to the first detection, and our human surveyor failed to find any outplanted TTF. If we retained this outlier in our analysis, there was no longer an effect of the surveyor on time to first detection (F = 1.989, *df* = 1, 10, *p* = 0.188). There was no effect of site on time to first detection in either analysis (outlier excluded: F = 2.114, *df* = 2, 10, *p* = 0.173; outlier included: F = 0.093, *df* = 2, 11, *p* = 0.912).

#### False negatives

Our human surveyor had a significantly greater probability of failing to detect outplanted TTF within a plot relative to our dog-handler team, if one or more outplants were present (χ2 = 4.89, *df* = 1, *p* = 0.027). Site did not influence false negatives (χ2 = 0.017, *df* = 2, *p* = 0.992).

#### False positives

Our dog-handler team recorded six alerts that could not be confirmed. The rate of false positives recorded for our dog-handler team did not differ between plots in which TTF had been outplanted or control plots (χ^2^ = 0.56, *df* = 1, 19, *p* = 0.454), suggesting that the presence of nearby TTF specimens does not influence Daisy’s false positive rate. We did, however, find that the rate of false positives depended on site (χ^2^ = 7.053, *df* = 2, 17, *p* = 0.029). Daisy showed higher false alerts at Nyora, a site with a low density of naturally occurring TTF, and the first site surveyed in our experiment. In contrast, our dog-handler team recorded no false positives at The Gurdies, the site with the highest density of naturally occurring TTF, and the final site that we visited. Combined, these results suggest that Daisy’s false positives are not associated with the abundance of TTF material.

### Natural *Hypocreopsis amplectens* specimens

Our human surveyor and dog-handler team performed equally when detecting naturally occurring TTF (χ2 = 1.017, *df* = 1, *p* = 0.313; human surveyor = 15; dog-handler team = 10; total = 18). In addition, our human surveyor identified four non-experimental TTF “scars” (i.e., evidence of past sporing bodies that can persist for several years) in two search plots. Still, our two teams performed equally when considering live specimens and scars together (χ2 = 2.795, df = 1, *p* = 0.095).

## Discussion

For the first time, we demonstrate that wildlife detection dogs enhance the detection of a rare and cryptic fungus, with important implications for the conservation of threatened fungi. After nine months of training on TTF odor, our dog-handler team out-performed our human surveyor, with greater detection rates, faster times to first detection, and less chance of failing to detect our target species when it was present (i.e., false negatives). Given the success of our WDD team in detecting what we consider is a relatively challenging fungal target – that is, an extremely rare and cryptic species with limited material available for training, a mycoparasite where scent profiles of both the target and host species were present in training materials, and a species with speculated low volatility (concentration of volatile organic compounds that emit odor, as discussed later in discussion) – we believe that our findings are extremely encouraging, and suggest that WDDs have great promise for aiding the conservation and management of other fungal species.

Several other studies have also shown that WDDs can outperform human surveyors:^19^ detecting a greater proportion of targets^30^^,^^31^^,^^32^^,^^33^ in less time.^32^^,^^33^^,^^34^ As dogs primarily sense via olfaction, they have a significant advantage over humans when searching for hidden or cryptic targets—under vegetation, for example.^35^ Daisy’s relatively high performance, compared to our human surveyor, is no doubt linked to the difficult nature of visual TTF detection, and the dense and complex habitat in which it occurs. Furthermore, we observed that our WDD-handler team caused less visible stress to sites relative to our human surveyor—Daisy is small, agile, and often searched without her handler immediately trailing her. Many fungi are likely difficult to detect, which may partially account for their taxonomic underrepresentation and lack of threat assessments.^26^ Therefore, WDDs may provide a low impact means of improving fungal conservation via enhanced survey efficiency and effectiveness.

Based on observations made during Daisy’s training and the current study, we suspect that TTF has extremely low volatility. Daisy most commonly needed to pass her nose close to TTF specimens for successful detection (<0.25 m)—her trainer indicated that on rare occasions Daisy had picked up on a scent from a few meters away and was working back toward the origin. This small detection distance meant that slow and fine-scale surveys were performed to maximize likelihood of detection. Past studies suggest that air-scenting is the most common search technique used by WDDs for detection.^18^ Indeed, the sensitivity of scat detection has been recorded as >75% when within 10 m,^36^ while for tortoises, the detection was possible at 0.5–60 m.^37^ We believe that Daisy’s success in searching for TTF in this study is therefore extremely encouraging, showing that WDDs may be incorporated in the conservation of fungal species, even if their volatility is low.

While this is the first documented case of WDDs assisting with fungal detection within a conservation context, dogs have previously demonstrated the ability to detect fungi for the primary production industry. Examples of their impressive utility for this purpose include the discrimination of truffles based on their ripeness.^38^ Our target species represented an equally challenging scenario for scent-based detection as TTF sporing bodies are partially composed of tissue from the fungal species that it parasitises.^39^ This “host” fungus also occurs throughout the local environment at densities much greater than TTF and, in fact, it is the primary visual indicator of suitable TTF habitat. Daisy demonstrated a clear ability to differentiate between TTF and its host throughout her training and our experiment, detecting scent unique to TTF sporing bodies, while ignoring the scent of host fungal tissue. This ability to discriminate between TTF and the host fungus is further evidenced by Daisy’s low false-positive rate, even though host fungal tissue was abundant throughout all experimental search plots.

On six occasions, Daisy’s handler communicated that she was showing a strong detection response, but no TTF specimens could be visually verified by our team. While this may be a true false alert, it is also possible that Daisy either detected a small TTF that we failed to visually confirm, or that Daisy was able to detect volatile compounds from TTF before it is visible (i.e., the detection of mycelia). This hypothesis is supported by studies where WDDs have been used to detect fungal material in pre-symptomatic trees infected with vascular wilt.^21^ Furthermore, incorrectly perceived false alerts by WDDs have been documented in studies of truffle^38^ and Antechinus,^40^ where positive identification was confirmed at a later date. In follow-up surveys conducted in 2023, we revisited Daisy’s unverified points of interest, but again found no evidence of TTF sporing bodies at these locations. In future, taking eDNA samples of wood or host material following a false alert could confirm whether Daisy is in fact detecting TTF before it becomes visible.

An advantage that our human surveyor had over Daisy was their ability to visually detect TTF “scars.” Although it may be possible to incorporate scar detection into Daisy’s future training (if they retain a distinct scent), at present, it appears that WDDs could fail to determine the presence of TTF if only scars were present at a site at a given time. This limitation could be overcome by including mixed teams of both WDD and humans when performing TTF surveys. Although, our WDD team’s performance was equal to the combined tally in our outplant experiment, each surveyor recorded unique detections. Therefore, the combined performance of both surveyors resulted in the highest number of detections overall, resulting in a 95% accuracy rate. This finding suggests that in the case of TTF detection, search teams that include WDDs and human surveyors will provide the most robust estimates of TTF abundance and will have the least chance of failed detection. Given that Daisy found a higher proportion of targets in a shorter time, working with WDD teams may result in shorter field days and less human effort, reducing the overall costs of the conservation program.

Establishing a WDD program *de novo* has high associated costs, however, working with established WDD teams can substantially reduce these costs.^41^ Although we worked with an established team, Daisy’s initial (pre-experimental) training required additional support from an experienced mycologist to confirm detections, however, this commitment decreased substantially post-training. Given that this was Daisy’s first TTF season, we anticipate that the dog-handler team’s species-specific search performance will improve with further training and experience. WDDs can discriminate trained target odors for at least 12 months without follow-up training on the target scent^42^^,^^43^; and this retention likely benefits from routine maintenance training on general odors (such as rubber) performed by established WDD teams.^44^ Indeed, one year after our experiment, Daisy successfully detected several new TTF specimens during surveys at new and previously visited sites (unpublished data). This followed minimal off-season maintenance training on the specific target odor (∼5 × 30-min sessions), which suggests ongoing training costs would be minimal when working with an established WDD team.

Here, we demonstrate that working with WDDs can be a cost-effective way to improve the detection of fungi and thus enhance their conservation. Conservation of fungi is critical, given our reliance on them for food, medicine and maintaining a healthy environment. We show that, relative to human effort, working with WDDs to detect rare and cryptic fungi can result in (i) a greater proportion of detections, (ii) faster times to first discovery and (iii) less chance of failing to detect specimens. As TTF represents an especially challenging fungal system, we anticipate that WDDs can be broadly integrated into fungal conservation efforts. Our research will no doubt enhance the continuing efforts to document and conserve an understudied kingdom that is threatened by continuing habitat loss, as well as a warming and drying climate.

### Limitations of the study

This work represents the first demonstration of the effectiveness of Wildlife Detection Dogs in surveying for an endangered fungus from a conservation context. For practical and logistical reasons (cost and training investment), we investigated the performance of a single experienced WDD relative to that of a single, highly skilled human (as COVID-19 restrictions in Australia meant that a larger team could not be assembled). While our experimental design would have benefited from the inclusion of additional WDDs and human surveyors, our study still demonstrates that a WDD can outperform our most skilled human surveyor when detecting a particularly challenging fungus. As such, despite these limitations, we believe our work is a valuable contribution that extends the applicability of WDDs to an often overlooked (from conservation and taxonomic perspectives) kingdom and will positively inform fungal conservation and management efforts.

## STAR★Methods

### Key resources table

**Table:** 

REAGENT or RESOURCE	SOURCE	IDENTIFIER
**Deposited data**		

Raw detection data	This study	https://doi.org/10.17632/69xry6fx2h.1

**Software and algorithms**		

R v4.3.1	R core Team^45^	https://www.r-project.org/
lme4	Bates et al.^46^	https://github.com/lme4
car	Fox and Weisberg^47^	https://github.com/cran/car
multcomp	Hothorn et al.^48^	https://github.com/cran/multcomp

### Resource availability

#### Lead contact

Further information can be requested via the lead contact, Michael Amor (michael.amor88@gmail.com).

#### Materials availability

This study did not generate any new reagents.

#### Data and code availability

All code and raw data used in this study have been uploaded to Mendeley Data: https://doi.org/10.17632/69xry6fx2h.1 and it is publicly available as of the date of publication. Any additional information required to reanalyse the data reported in this paper is available from the lead contact (michael.amor88@gmail.com) upon request.

### Experimental model and study participant details

Institutional Permission (IRC): Daisy’s involvement in this research was approved by the Zoos Victoria Animal Ethics Committee (approval #ZV20010). All *Hypocreopsis amplectens* samples were collected under Royal Botanic Garden Victoria’s Permit (10008918), provided by the Victorian Department of Energy, Environment and Climate Action.

#### Materials and methods

##### Detection dog training and human surveyor experience

Daisy is a 5-year-old, desexed female Lagotto Romagnolo with four years’ experience surveying for multiple target odours in field conditions, including avifauna carcasses, freshwater turtle nests, and various training odours including Kong® rubber. Her TTF detection training, led by NJR, commenced in November 2021 at Healesville Sanctuary, Victoria, Australia; nine months prior to our experiment. MDA provided TTF-specific guidance during Daisy’s training. Experience working within a particular habitat is important to search performance,^49^ therefore, Daisy’s training also included *in situ* sessions in similar habitat to where TTF occurs. Our human surveyor, SB, is highly skilled at detecting TTF. Indeed, as of 2023, she has directly detected more TTF specimens than any other human. Details of Daisy’s training and our human surveyors’ experience are presented in the associated supplementary methods.

##### Outplant specimen collections and storage

As a mycoparasite, the entire fungus is restricted to growing on above-ground decaying wood—this allowed us to collect small branches hosting TTF for our experiment. Three ∼20 cm long branches hosting TTF sporing bodies were collected from two sites in Victoria, Australia: French Island (*n*=1) and The Gurdies (*n*=2) to act as experimental outplants. These outplants were kept in glass spaghetti jars with silicone o-rings and, outside of surveying hours, were stored at 4°C to limit decay and contamination. Outplants were always handled with forceps and fresh nitrile gloves.

Given the rarity of TTF, we used the three outplant specimens across our entire experiment (which spanned three consecutive weeks, see Experimental methods). On the first search day of each week, we tested whether Daisy was still able to detect these outplants to ensure that long-term storage had not led to decay or altered the scent profile. To do this, we placed all three outplants in natural vegetation along a ∼15 m transect that Daisy searched. In all cases, Daisy was able to detect all three specimens after five minutes of searching, thus our storage conditions were suitable and reusing these specimens did not compromise our experiments. For consistency, our human surveyor was also allowed to investigate these specimens at this time.

### Method details

We performed a total of 21 searches across three sites (seven searches each at Nyora, Grantville and The Gurdies) situated in south-east Victoria, Australia, over three consecutive weeks, from 18 July to 04 Aug 2022. Our searches were conducted within and around TTF populations that we identified in 2020–2021. All sites had complex and dense (but varied) vegetation (e.g., Figure 1A). Nyora and Grantville are known to have a very low density of TTF (between 0–5 specimens observed in 2021–2023), while The Gurdies is a relatively high-density site (between 10–35 specimens observed in 2021–2023). We focused our searches on one site per week to minimise the accidental spread of invasive fungi among study sites.

For each site, we set up seven ∼15 × 15 m search plots (225 m^2^) marked by flagging tape one day before the first experimental searches commenced. During this time, no members of the experimental search teams were present. We noted the GPS coordinates of each corner of each plot and completed visual surveys for TTF for 15–30 minutes and recorded any naturally occurring TTF.

Prior to each survey, MDA (not the human surveyor) haphazardly placed 0–3 of the TTF outplants within the pre-marked plots up to 0.75 m above ground level in locations consistent with natural TTF occurrence. Our experiment had five control plots, that received no outplanted TTF specimens; and 16 positive plots, that received ≥1 outplanted TTF specimens, that were randomly assigned across our 21 plots. The number of outplants did not differ among sites (F=0.11, *df=*2, p=0.90). Each site had at least one control plot which we used to test whether the presence or absence of TTF outplants influenced Daisy’s false alerts (indicating TTF was present when it was not). To ensure that human odour was evenly distributed throughout each plot, the team member responsible for setting up the experiment also spent 15 minutes moving haphazardly throughout the plot and stopped and handled (with gloves) natural plant material to ensure that Daisy was responding to outplanted TTF, rather than human odour.

To allow for thorough searching, both the dog-handler team and our human surveyor were given up to 45 minutes to search each plot, starting from the same corner. The dog-handler team searched first to avoid the influence of scent trails left by our human surveyor. We measured the detection of experimental outplants and the time it took each surveyor to detect them. Positive identification by Daisy was based on her trained alert behaviour which was communicated by her handler, NJR, to the data scorer, MDA. We scored a false positive when Daisy’s handler communicated that Daisy was displaying alert behaviour, but no outplant had been placed at that location, and no naturally occurring TTF could be detected after 20 minutes of searching. We did not score false positives (i.e., misidentifications) for our human surveyor, as these were rare, and became increasingly less frequent as the experiment progressed due to a reluctance for making an error. False negatives were scored when a surveyor failed to detect an outplant in a plot that had received at least one.

From this experimental data, we calculated the proportion of experimental outplants detected by each observer by dividing the number of outplanted TTF specimens detected by the total number that had been outplanted in each plot. We also calculated a combined tally by summing the number of unique TTF outplants found by our WDD-team and our human surveyor (or those found by both) and dividing by the total number of outplants received by each plot. We scored false negatives as a binary response variable: values of 0 indicate failed detection in plots that received outplants; values of 1 indicate that at least 1 outplant was detected in a plot that had received outplants. Daisy’s false positives were also scored as a binary response variable: values of 0 indicate no false alerts were made during a given search; values of 1 indicate that a false alert was made during the search. We also noted any identifications of naturally occurring TTF within each plot, as well as the time it took to detect these specimens.

### Quantification and statistical analysis

#### Detection of experimental outplants

For our outplant experiment, we assessed differences in surveyor success using several response variables: (i) the proportion of outplants detected; (ii) time to first detection; (iii) the rate of false negatives; and (iv) the rate of false positives made by the WDD-team. We performed analyses (i–iii) using Generalised Linear-Mixed Models (GLMMs) with either gaussian (i–ii) or binomial (iii) error distributions and used Maximum Likelihood for estimating our fixed effects. GLMMs included site and surveyor as categorical fixed effects, and plot(site) as a nested random effect to account for the non-independence of plots that were searched by our surveyors. For GLMMs we only used the data from plots where TTF had been outplanted. For model (i) we included three levels for our predictor of surveyor: WDD-team alone, human surveyor alone, and their combined tally; for (ii–iii) we excluded the combined tally due to issues with multicollinearity. We analysed (iv) using Generalised Linear Models (GLMs) with binomial error distributions using data from only the WDD-team surveys from all plots (those with and without outplants). For these GLMs, outplant presence and site were categorical fixed effects. For all analyses, we removed non-significant interactions between our fixed effects from the final models and performed Tukey’s post-hoc tests to investigate significant main effects where appropriate. All analyses were performed in R^45^ using the base environment as well as packages ‘lme4’,^46^ ‘car’^47^ and ‘multcomp’.^48^

#### Naturally occurring individuals

We compared the number of naturally occurring TTF sporing bodies detected by our WDD-team and human surveyor using linear-mixed models where surveyor was a categorical fixed effect and plot was a random effect. Site was not included in this analysis, as all naturally occurring TTFs were found at the Gurdies, except for one plot at Nyora.

## Supplemental information

Document S1. Surveyor training and experience
