# Supplementary material for: On the trail of a critically endangered fungus: A world-first application of wildlife detection dogs to fungal conservation
Source: iScience. 2024 Apr 29;27(5):109729. doi: 10.1016/j.isci.2024.109729 (PMC11123565; doi:10.1016/j.isci.2024.109729)
Supplement: Document S1. Surveyor training and experience [file mmc1.pdf]

iScience, Volume *27*

Supplemental information

On the trail of a critically endangered fungus: A world-first application of wildlife detection dogs to fungal conservation

Michael D. Amor, Shari Barmos, Hayley Cameron, Chris Hartnett, Naomi Hodgens, La Toya Jamieson, Tom W. May, Sapphire McMullan-Fisher, Alastair Robinson, and Nicholas J. Rutter

Wildlife detection dog training

Hypocreopsis amplectens (tea-tree fingers; TTF) tissue samples (n = 5) were collected from The Gurdies and French Island (Victoria, Australia) into sterile glass culture vials using gloves and sterilized forceps. These specimens were first sub-sampled for DNA isolation for a concurrent project (Amor et al. in prep) and the remaining tissue (5–10 mm2) was stored in a frost-free freezer (-20°C) for use in training of our wildlife detection dog, Daisy, to search for and identify TTF. Handling of all TTF samples and training aids was always conducted with gloves and forceps.

Daisy was initially introduced to TTF odor in controlled indoor conditions using defrosted material inserted into clean Polyvinyl Chloride (PVC) 'scent pot' containers fitted with mesh caps. These scent pots allowed odour diffusion, but prevented physical access to samples. Training exercises included up to ten scent pots that were attached to a timber 'scent board', enabling presentation of both target and non-target material during training (Rutter et al., [14, 45]—references in main text). In these trails, Daisy was exposed to sub-samples of five specimens from two sites that ranged in size from approximately 10–25 mm2. Daisy was trained to alert her handler of the presence of TTF within these scent pots by sitting and looking to her handler, without physically interacting with the specimen.

Secondary training was conducted in dense tea-tree dominated forest within the Coranderrk Nature Conservation Reserve that was representative of known *H. amplectens* habitat. First, Daisy was tasked to search for a familiar training odour (Kong® rubber cut into approximately 3–4 mm3) in double blind search assessments was established in tea-tree forest. Once a mean sensitivity score of 90% was reached, training with TTF tissue commenced.

This training initially focused on searching for defrosted TTF in simple search scenarios where target locations were known to NR, before progressing to double blind searches of up to 15m x 15m (225 m2) for up to 45 minutes. The volatility of TTF odour was estimated to be low, with Daisy most often needing to pass within approximately 30–50 cm of specimens to detect them. Daisy's training therefore emphasised thorough searching of potential host branches from ground level up to approximately one meter high.

Where possible, fresh and defrosted samples were prioritised for use in training to provide Daisy with a realistic target odour profile. However, due to the scarcity of training material, training also included the use of Getxent® odour collection tubes impregnated with TTF odour for 24 hours. Observations made during these training exercises indicated that Daisy could detect these odour tubes from a greater distance than defrosted TTF material. In contrast, impregnating Getxent® tubes for eight hours and cutting these in appeared more similar in volatility to defrosted material.

Finally, training was undertaken at a naturally occurring TTF population on private land in Launching Place, Victoria. This allowed us to ensure that Daisy could generalise her detection to live, novel TTF specimens growing in situ, which may have different odour profiles to the defrosted training samples that were collected from different sites (separated by ~80 kms). We also conducted scent board training on novel, non-experimental *H. amplectens* samples from French Island and The Gurdies to ensure confident detection and odour generalisation across samples collected from the known distribution of *H. amplectens*.

Human observer training and experience

Shari has experience working across various field-based projects, and, prior to commencing our field experiments, she spent one season conducting traditional visual searches for *H. amplectens*. As of 2024, she has detected more *H. amplectens* sporing bodies than any other person.
